# ScInfoVAE: interpretable dimensional reduction of single cell transcription data with variational autoencoders and extended mutual information regularization

**DOI:** 10.1186/s13040-023-00333-1

**Published:** 2023-06-10

**Authors:** Weiquan Pan, Faning Long, Jian Pan

**Affiliations:** 1 School of Mathematics and Statistics, Yulin Normal University, Yulin, China; 2 School of Computer Science and Engineering, Yulin Normal University, Yulin, China

**Keywords:** Unsupervised clustering, Deep neural network, Variational autoencoder, Mutual information, scRNA-seq data

## Abstract

Single-cell RNA-sequencing (scRNA-seq) data can serve as a good indicator of cell-to-cell heterogeneity and can aid in the study of cell growth by identifying cell types. Recently, advances in Variational Autoencoder (VAE) have demonstrated their ability to learn robust feature representations for scRNA-seq. However, it has been observed that VAEs tend to ignore the latent variables when combined with a decoding distribution that is too flexible. In this paper, we introduce ScInfoVAE, a dimensional reduction method based on the mutual information variational autoencoder (InfoVAE), which can more effectively identify various cell types in scRNA-seq data of complex tissues. A joint InfoVAE deep model and zero-inflated negative binomial distributed model design based on ScInfoVAE reconstructs the objective function to noise scRNA-seq data and learn an efficient low-dimensional representation of it. We use ScInfoVAE to analyze the clustering performance of 15 real scRNA-seq datasets and demonstrate that our method provides high clustering performance. In addition, we use simulated data to investigate the interpretability of feature extraction, and visualization results show that the low-dimensional representation learned by ScInfoVAE retains local and global neighborhood structure data well. In addition, our model can significantly improve the quality of the variational posterior.

**Supplementary Information:**

The online version contains supplementary material available at 10.1186/s13040-023-00333-1.

## Introduction

In recent years, single-cell RNA sequencing (scRNA-seq) has become a hot topic in the field of biology as sequencing technologies continue to improve and cellular research deepens. Single cell RNA sequencing is a new technology that enables high-throughput sequencing of the genome at the level of individual cells. Traditional high-throughput RNA sequencing is based on the whole tissue and the genomic information is averaged across the group; single-cell RNA sequencing provides data representing the genetic information of individual cells, which can be used to study genetic heterogeneity between cells of the same phenotype and to discover their specific biological functions [1]. An important function of single-cell sequencing data is to differentiate between cell types, i.e. to build clustering models based on single-cell sequencing data that can be used to cluster cells with similar gene expression patterns into the same cell type, to infer cell function and to gain an understanding of the correlation between disease and genomic features [2]. If a more accurate and unbiased clustering of cells could be achieved, this would have a huge impact in areas such as oncology, gene expression and immunology [3].

Currently, most cell clustering methods are proposed based on traditional RNA sequencing data, and although they can be used on scRNA-seq data, scRNA-seq data have characteristics that clearly distinguish them from traditional high-throughput RNA data, such as large data volume, high dimensionality and having excessive noise. The direct use of clustering algorithms developed based on tissue RNA-seq data on scRNA-seq data has significant constraints [4], so the design and construction of clustering algorithms applicable to the feature of single-cell data has become a hot subject in the field of single-cell analysis.

The most critical step in the data analysis of scRNA-seq is to group cells belonging to the same cell type according to the gene expression pattern that can make them similar within the group and different between groups. However, the expression data of scRNA-seq makes cell clustering difficult due to the following reasons:

1. Compared with traditional scRNA-seq, one can obtain the expression amount of each gene in each cell with higher precision and wider dimensions to use for more downstream personalized analysis.
2. As each gene expression in each cell is to be sequenced, but not all genes in all cells can be transcribed and expressed in real life. Therefore, the expression data of scRNA-seq is a sparse matrix and the average sparsity can be as high as 50 percent, i.e., many genes in a cell of the gene expression are zero, even 50 percent of them are zero.
3. A project can obtain tens of thousands to hundreds of thousands of cell samples and the number of genes is usually tens of thousands, such a high dimensional data set is difficult to analyze the differences in gene expression patterns between cell types.

According to the characteristics of scRNA-seq data, the scRNA-seq data first needs to be dimensionalized.Some linear dimension-reduction method has been favored by researchers such as principal component analysis (PCA). In addition, there are non-linear methods such as uniform manifold approximation and projection (UMAP) and t-distributed stochastic neighbor embedding (t-SNE) to reduce dimension. After the rise of neural network, there are many methods of dimensionality reduction based on neural network [5–7]. In addition, the dropout events in scRNA-seq data may make the classic dimensionality reduction algorithm unsuitable. Pierson and Yauet al. 2015[8] modified the factor analysis framework to solve the dropout problem and provided a method zero-inflated factor analysis (ZIFA) based on an additional zero-inflation modulation layer for reducing the dimension of single-cell gene expression data. Compared with the above two linear methods, employing the zero-inflation model can give ZIFA more powerful projection capabilities but will pay a corresponding cost in computational complexity.

Existing non-linear dimensionality reduction methods still have limitations such as lack of robustness to random sampling, inability to capture the global structure while focusing on the local structure of the data, sensitivity to parameters and high computational cost. Currently, the scale of scRNA-seq data has reached tens of thousands or even millions of cells, and deep learning techniques, as widely used non-linear transformation methods, are applicable to dimensionality reduction of large-scale scRNA-seq data. In deep learning methods, the use of Zero-inflated Negative Binomial (ZINB) model instead of the Mean Square Error (MSE) objective function commonly used in autoencoders can better handle the large number of “false zeros” in scRNA-seq data. It can better characterize scRNA-seq data, and explicitly optimize clustering when dimensionality reduction is performed [9]. For example, the scDeepCluster model [10] combines the ZINB model and deep learning’s AutoEncoder trained together to map scRNA-seq data to a low-dimensional space on which the clusters are clearly partitioned using KL (Kullback-Leibler ) divergence constraints, improves the computational efficiency of scRNA-seq data analysis.In addition, to further improve the accuracy of feature learning, some novel deep clustering algorithms have recently been proposed [11] that unify the learning of feature representations and class labels using a joint training strategy, which involves training the network parameters by making full use of the clustering results to output optimal feature representations, class centres, and class labels of cells. For example, Chen et al [12] proposed scziDesk, a joint training method combining fuzzy clustering and autoencoder based on scDeepCluster, which combines K-means with AutoEncoder and uses Soft K-means as the objective function to substantially improve the clustering accuracy of spherical cluster data. The method extracts low-dimensional features via the AutoEncoder in the first training, and adds the Soft K-means objective function to fine-tune the model in the second training to further improve accuracy. However, it is difficult to obtain the optimal solution during the second training, and there is a problem of gradually increasing and decreasing accuracy, which makes the training process unstable. Another major problem is the poor accuracy of clustering non-spherical data. Ciortan, M., Defrance, M.[13] investigated a method for extracting features using graph autoencoder networks that is robust to input down-sampling, insensitive to input data outliers, but requires some computational time for input data composition [13]. Grønbech C.H. et al proposed a new method, scVAE [14], based on Variational Autoencoder (VAE) [15], for the analysis of scRNA-seq data. It bypasses the process of data pre-processing and allows for robust estimation of expected gene expression levels and potential representations for each cell by using raw count data as input. Zhao, S et al.[16] found that the existing objective function of VAE may lead to inaccurate amortized inference distributions of the inferred data by the autoencoder and that the variational autoencoder tends to ignore potential hidden layer variables. They proposed the InfoVAE model that increases the input data and the hidden space data, which increases the regular terms of the mutual information metric. The InfoVAE model can significantly improve the quality of the variational posterior probability distribution of the variational autoencoder [16].

In summary, our contributions can be summarized as follows: (1)We propose an efficient training framework that integrates InfoVAE and ZINB, leveraging autoencoders for clustering scRNA-seq data. The proposed method produces competitive results on both simulated and real datasets than other similar deep-learning approaches, robust to changes in input parameters and flexible to allow the integration any suitable clustering algorithm. (2)It is a challenge for dimension reduction to interpret structure in scRNA-seq data . Existing algorithms are either not able to uncover the clustering structures in the data or lose global information such as groups of clusters that are close to each other. ScInfoVAE is able to capture and visualize the low-dimensional structures in scRNA-seq data . Simulation results demonstrate that low-dimensional representations learned by ScInfoVAE preserve both the local and global neighbor structures in the data. In addition, ScInfoVAE is robust to the number of data points and learns a probabilistic parametric mapping function to add new data points to an existing embedding.

## Methods

**Fig. 1:**
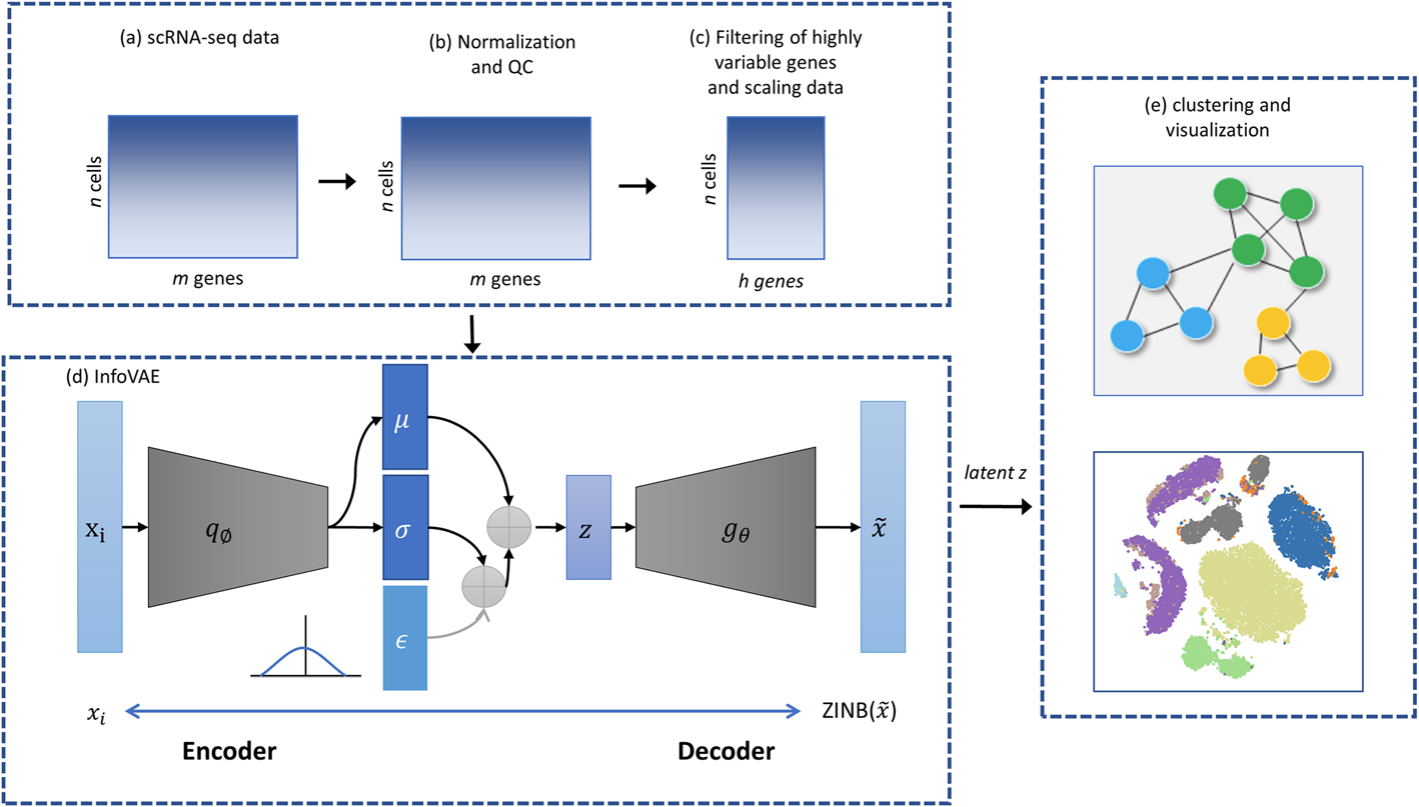
Workflow of clustering based on InfoVAE. The network is trained by both clustering loss and reconstruction loss

The deep clustering algorithm is divided into two separate processes of feature learning and cluster partitioning. The former part does not necessarily learn the feature representation that is most suitable for cluster partitioning, thus affecting the clustering performance. With the joint mutual information variational autoencoder (InfoVAE) and ZINB training strategy, the deep clustering problem can be described as follows: the original scRNA-seq dataset is defined as $D_n = \{x_1, x_2,\cdots , x_n\}$ where $x_i$ represents the *i*-th cell and *n* is the number of cells; the center of the clusters are $R_n = \{r_1, r_2, \cdots , r_k\}$, where *k* is the number of cell classes. The aim is to reduce dimension and cluster zero-inflated single-cell data. The Workflow of InfoVAE-based deep clustering is shown in Fig. 1a, where the scRNA-seq dataset was first preprocessed using SCANPY [17] to remove genes with “non-zero” expression below $1\%$ in all cells and to remove cells with “non-zero” expression below $1\%$ in all genes. The data were then normalised using a $\log (x+1)$ transformation and Quality Control(QC) is performed using SCANPY (Fig. 1b). The 500 genes with the highest variance (Fig. 1c) were identified and retained, and the data were centered and the value of each entry in the gene expression matrix was subtracted from the mean expression value of the cell in which it was located, making it easier to solve the covariance matrix with a variance of 1 between cells. The SCRNA data were pre-trained using InfoVAE combined with the ZINB model, called ScInfoVAE. The training process is then optimised jointly with the clustering objective function (Fig. 1d) and finally the embedded feature vectors are extracted and the latent feature vectors are clustered for analysis and visualisation (Fig. 1e). K-means and spectral clustering methods are used in this paper.

### InfoVAE

InfoVAE (Mutual Information in Variational Autoencoders) consists of two parts: the encoder and the decoder. The encoder is defined as: $f_{\varphi } X \rightarrow Z$ where $X_{p_{data}}(x)$ is the input raw data and *Z* is the reduced-dimensional data; $\varphi$ is the set of encoder network parameters. The decoder is defined as $g_{\theta }: Z \rightarrow \widetilde{X}$ where $\theta$ is the set of decoder network parameters. The dimensionality of the latent space *Z* is usually smaller than that of the data layer, so it can be used to learn a compressed representation of raw data. The reconstructed representation $\widetilde{X}$ must be as close as possible to the input data *X*. Here we defind $q(z_i|x_i,\varphi )$ as the deep neural network encoder, $p(x_i|z_i, \theta )$ as the deep neural network decoder, and $z_i$ as the latent space sample encoded by $q(z_i|x_i,\varphi )$. The loss function of the autoencoder is to minimize the reconstruction loss. The dimensional reduction is achieved by reducing the dimensionality of the latent layer feature space. However, if the autoencoder is trained with sufficient freedom, the reconstruction loss becomes small, reaching 0 when the network is deep enough (causing over-fitting). The variational auto-encoder incorporates some slight modifications to the autoencoder, and by encoding the input data as normally distributed data rather than as a number of points, over-fitting is effectively avoided. The generic form of the variational auto-encoding objective function [15] is defined as:

1 $$\begin{aligned} L_{V A E}=-\lambda D\left( q_{\phi }(z) \Vert p(z)\right) + \mathbb {E}_{p_{\text{ data } }(x)} \mathbb {E}_{q_{\phi }(z \mid x)}\left[ \log \left( p_{\theta }(x \mid z)\right) \right] . \end{aligned}$$

where $D \ge 0$ is a distance formula that measures the difference between two probability distributions, and $D(q||p) = 0$ when $p = q$ and $\lambda >0$ is a scaling factor. The traditional VAE solution process suffers from two major drawbacks. The first drawback can be categorized as being that the mutual information of the latent variable *z* and the observed variable x is too small. The second drawback can be categorized as the fact that the approximate posterior distribution $q_{\phi }(z|x)$ of the latent variable *z* will never converge to the true posterior distribution $p_{\theta }(z|x)$[18], even though it is possible to maximize the Mutual Information of the latent variable *z* and the observed variable *x*. To address these two issues, the mutual information $I_q(x; z)$ of the hidden variable *z* and the observed variable *x* is added to Eq. (1)as a regularization term to improve the generalization of the model, where $\lambda$ and $\alpha$ are the conditioning parameters, as shown in Eq. (2).

2 $$\begin{aligned} L_{\text{ InfoVAE } }=-\lambda D\left( q_{\phi }(z) \Vert p(z)\right) + \mathbb {E}_{p_{\text{ data } }(x)} \mathbb {E}_{q_{\phi }(z \mid x)}\left[ \log \left( p_{\theta }(x \mid z)\right) \right] +\alpha I_{q}(x ; z). \end{aligned}$$

Based on the derivation of reference [18], the objective function of Eq. (2) can be modified for programming implementation, as shown in Eq. (3)

3 $$\begin{aligned} L_{I n f o V A E}=\mathbb {E}_{p_{g_{\theta }(x)}} \mathbb {E}_{q_{\phi }(z \mid x)}\left[ \log \left( p_{\theta }(x \mid z)\right) \right] -(1-\alpha ) \mathbb {E}_{p_{g_{\theta }(x)}} D_{K L}\left( q_{\phi }(z \mid x) \Vert p(z)\right) \nonumber \\ - (\alpha +\lambda -1) D_{K L}\left( q_{\phi }(z) \Vert p(z)\right) . \end{aligned}$$

### Reconstructing the loss function

Consider the second term of $\mathbb {E}_{q_{\phi }(z \mid x)}\left[ \log (p_{\theta }(x|z))\right]$ which can be substituted in the autoencoder in the form of Eq. (4)

4 $$\begin{aligned} L_{V A E}=-\lambda D\left( q_{\phi }(z) \Vert p(z)\right) +\mathbb {E}_{p_{\text{ data } }(x)} \mathbb {E}_{q_{\phi }(z \mid x)}\left[ -\frac{\Vert x-g(z)\Vert ^{2}}{2 c}\right] . \end{aligned}$$

where the constant *c* is a regularization parameter weighing the left and right terms, with a larger *c* indicating a larger variance in the decoder and a greater tendency towards the regularization term and $z = f_{\varphi }(x_i)$ is the latent variable.

The effect of the encoder is to encode the high-dimensional input *X* into a low-dimensional latent variable *Z*, thus forcing the neural network to learn the features of the most useful information. The purpose of the decoder is to recover the latent variable *Z* in the latent layer to its initial dimension. The optimal outcome is that the output of the decoder is a perfect or close approximation of the original input, i.e. $X \approx \widetilde{X}$. The difference between the original input and the reconstruction is referred to as the reconstruction error. In order to learn the best encoding and decoding, the goal of the autoencoder is to minimize the reconstruction error, so the reconstruction loss function of the autoencoder is expressed in Eq. (5):

5 $$\begin{aligned} L_{r e c}=\left\| x_{i}-g_{\theta }\left( f_{\varphi }\left( x_{i}\right) \right) \right\| _{2}^{2}. \end{aligned}$$

The scRNA-seq data are highly sparse, in the sense that there are very many zeros (zero inflation). It is crucial to make reasonable assumptions about the distribution of the data. For the reads count data of scRNA-seq, the most common normal distribution is not reasonable. Firstly, the normal distribution describes continuous data, whereas the reads count data is discrete; secondly, the reads count data can only take values that are nonnegative integers. After numerous attempts, the use of the ZINB distribution [9] to process the output $\widetilde{X}$ and then calculate the mean square error was found to be more capable of differentiating cell types than a typical autoencoder using the mean square error (MSE) loss function alone. This suggests that the use of a computational model in the autoencoder to process the output data is necessary to derive features from the scRNA-seq data.

Since zero values in gene representation data can result from both genes that are not expressed during the biological process (called True Zero) and from technical losses during sequencing (called False Zero or Dropout Zero), a zero-inflation is added to the negative binomial NB (without zero-inflation) model. The zero-inflated negative binomial distribution (ZINB) is used to model the autoencoder output data and calculate the reconstruction loss.

6 $$\begin{aligned} \text {NB}\left( X^{\text{ count } } \mid \mu , v\right) =\frac{\Gamma \left( X^{\text{ count } }+v\right) }{X^{\text{ count } } ! \Gamma (v)} \times \left( \frac{v}{v+\mu }\right) ^{v}\left( \frac{v}{v+\mu }\right) ^{X^{\text{ count }}}. \end{aligned}$$

7 $$\begin{aligned} \text {ZINB}\left( X^{\text{ count } } \mid \pi , \mu , v\right) =\pi \delta _{0}\left( X^{\text{ count } }\right) + (1-\pi ) \text {NB}\left( X^{\text{ count } } \mid \mu , v\right) . \end{aligned}$$

where $X^{\text {count}} = g_{\theta }(f_{\varphi }(x_i))$ denotes the decoder output data of the autoencoder, $\pi$ denotes the ratio of zero values, and $\mu , v$ are the parameters of the negative binomial distribution. Thus Eq. (5) can be written as

8 $$\begin{aligned} L_{\text {rec}}=\left\| x_{i}-\text {ZINB}\left( g_{\theta }\left( f_{\varphi }\left( x_{i}\right) \right) \right) \right\| _{2}^{2}. \end{aligned}$$

### Deep clustering with KL divergence

Optimizing the autoencoder and extracting the latent embedding features is challenging. However, unlike supervised learning, we cannot train our deep networks with labelled data. There is a considerable literature, which proposes the use of soft clustering in the auxiliary target distribution to assign feature vectors as a training iteration to obtain a more clustering-friendly feature representation [19–21].

The Student-t distribution can be used to estimate the overall sample mean that is normally distributed and has unknown variance based on a small sample, and we will design the auxiliary target distribution based on the Student-t distribution. The objective function $L_{KL}$ is therefore defined as the KL divergence between the soft distribution $q_i$ and the auxiliary distribution $p_i$, as shown in Eq. (9).

9 $$\begin{aligned} L_{K L}=K L(P \Vert Q)=\sum _{i} \sum _{j} p_{i j} \log \frac{p_{i j}}{q_{i j}}. \end{aligned}$$

where $p_{i j}$ is the cluster optimisation target distribution. By squaring $q_i$ to calculate $p_i$, we expect to bring the data points closer to the cluster centers and improve the cohesion of the clusters. The data representation is optimized by learning a high confidence assignment such that the sum of the projected distances of all data points from the nearest cluster center $r_i$ is minimized, as shown in Eq. (10).

10 $$\begin{aligned} p_{i j}=\frac{q_{i j}^{2} / \sum _{j} q_{i j}}{\sum _{j^{\prime }}\left( q_{i j^{\prime }}^{2} / \sum _{j^{\prime }} q_{i j^{\prime }}\right) }. \end{aligned}$$

where $q_{ij}$ is the probability of assigning data point *i* to cluster *j* (i.e. soft assignment). The similarity between the latent embedding $z_i$ and the cluster center $r_j$ is measured using Student’s t-distribution as a kernel, as shown in Eq. (11).

11 $$\begin{aligned} q_{i j}=\frac{\left( 1+\left\| z_{i}-r_{j}\right\| ^{2} / \alpha \right) ^{-\frac{\alpha +1}{2}}}{\sum _{j^{\prime }}\left( 1+\left\| z_{i}-r_{j^{\prime }}\right\| ^{2} / \alpha \right) ^{-\frac{\alpha +1}{2}}}. \end{aligned}$$

where, for $x_i \in X$, its low-dimensional embedding vector $z_i = f_{\varphi }(x_i) \in Z$; $\alpha$ is the degree of freedom of the Student-t distribution. Since $\alpha$ cross-validation on the validation set cannot be performed during unsupervised learning, $\alpha$ is set to 1. In summary, the final objective function is shown in Eq. (12).

12 $$\begin{aligned}&L_{\text{ SCInfoVAE }}=-\lambda D\left( q_{\phi }(z) \Vert p(z)\right) +&\nonumber \\&\mathbb {E}_{p_{\text{ data } }(x)} \mathbb {E}_{q_{\phi }(z \mid x)}\left[ -\frac{\left\| x-Z I N B\left( g_{\theta }\left( f_{\varphi }(x)\right) \right) \right\| ^{2}}{2 c}\right] +\gamma L_{K L}.&\end{aligned}$$

where $\lambda$ and $\gamma$ are regulating parameters to balance the training process for each loss function.

## Experiments and results

In this section, we construct a deep clustering model for scRNA-seq data using the method proposed in this paper. We tested the clustering performance on 15 real datasets and the ability of ScInfoVAE to learn low-dimensional representations that preserve local and global neighborhood structure on simulated datasets.

### Evaluation criteria

The real scRNA-seq datasets with actual class labels used in this paper was evaluated for clustering performance using four metrics. Adjusted Rand Index (ARI) score [22], Normalised Mutual Information [23] and internal score(Silhouette) [24] and Calinski Harabasz [25]. For all metrics, the higher the value, the better the performance.

ARI is commonly used in cluster analysis to measure the degree of agreement between two data partitions. The ARI will be used to quantify the agreement between a reference and a predicted clustering on the scale $[-1,1]$, with score of 1.0 denoting perfect agreement. However, as there might be multiple equally valid/plausible/useful partitions , the outputs generated by a single algorithm is evaluated against all the available reference labellings and the maximal similarity score is reported.

Normalized Mutual Information (NMI) is a measure used to evaluate network partitioning performed by community finding algorithms. It is often considered due to its comprehensive meaning and allowing the comparison of two partitions even when a different number of clusters.

The silhouette score is based on the principle of maximum internal cohesion and maximum cluster separation. In other words, we would like to find the number of clusters that produce a subdivision of the dataset into dense blocks that are well-separated from each other. In this way, every cluster will contain very similar elements and, selecting two elements belonging to different clusters, their distance should be greater than the maximum intra-cluster one.

The Calinski-Harabasz index (also known as the Variance Ratio Criterion) is calculated as a ratio of the sum of inter-cluster dispersion and the sum of intra-cluster dispersion for all clusters (where the dispersion is the sum of squared distances). It is most commonly used to evaluate the goodness of split by a K-Means clustering algorithm for a given number of clusters.

### Datasets

We collected 15 published scRNA-seq datasets. These datasets were generated from representative sequencing platforms such as the 10X genomics platform, the Drop-seq platform, and the SMART-seq2 platform. Class labels for these datasets were obtained using the computational methods from the cited papers. Specific details of the datasets are described in Table 1 of Supplementary Materials.

### Experiments and analysis

In this section, we perform a clustering test on the dataset in Table 1 of Supplementary Materials. We used the cell type information provided by the authors of the dataset. They identified cell types experimentally or by other biological methods, and these cell type labels can be considered as benchmarks for clustering accuracy. We then applied our method to obtain clustering results and analysed them using an ARI, where higher ARI values are associated with better clustering.

The lower the starting RNA amount in single-cell sequencing, the lower the gene expression correlation of technical replicate samples. Therefore, after generating a standardised gene expression profile, a very important step is to assess the technical variability. ScziDesk proposes an ablation study in which the raw data is pre-processed to extract a small number of highly variable genes and eliminate outliers, which not only reduces the dimensionality of the data and increases the training speed, but also significantly improves the clustering performance. It is therefore necessary to perform pre-processing of the raw data by SCANPY [17] before inputting it into the model for training. Similar to ScziDesk, the 500 genes with the highest discrepancies were retained as model input.

**Fig. 2:**
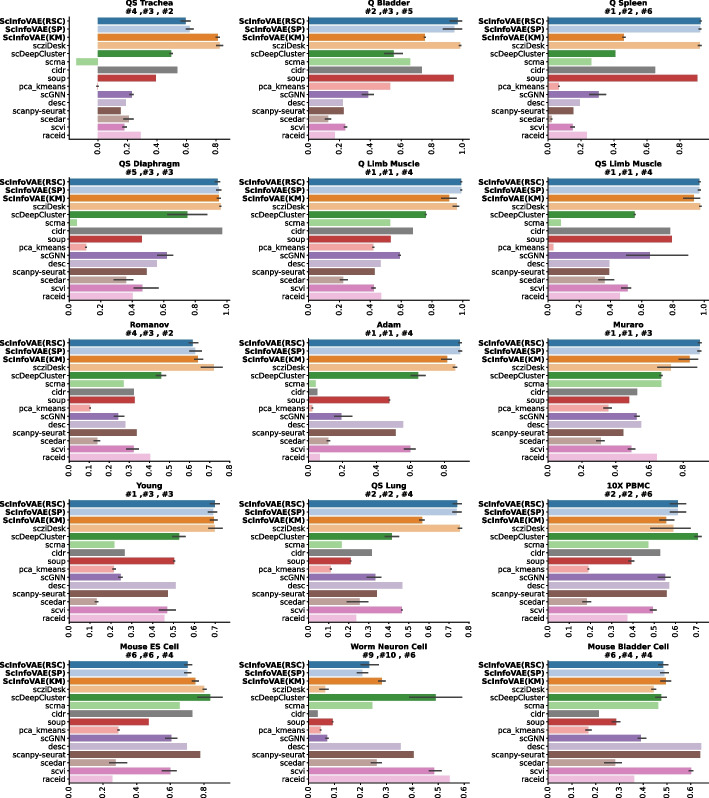
Dataset-level analysis of real scRNA-seq data on ARI scores. The dataset annotations (e.g. #1) indicate the ranking of ScInfoVAE, respectively, with K-means-Spectral-clustering and RSC clustering on each analyzed dataset

The dataset was trained and tested independently using various methods from Table 2 in Supplementary Materials. Among them ScDeepCluster, SCVI, scziDesk, etc. all use the default parameter settings of the original author’s source code and they are not optimised. ScInfoVAE uses the same denoising autoencoder model as ScDeepCluster, and we set the size of the autoencoder to $d - 500 - 256 - 256 - 10$ , where *d* is the size of the input data. Using Eq. (3), pre-train the mutual information-based autoencoder. The final optimization is performed using Eq. (12). The pre-training used 600 epochs and the optimisation process used 500 epochs. We use Adam optimizer[26] as the optimizer, and the learning rate is set as $10^{-3}$ in pre-training and $10^{-4}$ in the fine-tuning stage. After training and extracting the feature vectors, the ARI results were obtained using the K-means, SpectralClustering of the scikit-learn python package and Robust Spectral Clustering(RSC) for Noisy Data(Bojchevski, Matkovic, and Günnemann 2017[27]), respectively. We performed all methods 5 times and reported the average results to prevent extreme cases. Of these, for all the methods that used the K-means algorithm to generate clustering assignments, we initialised five times and selected the best solution, and the results are shown in Fig. 2. ScInfoVAE(KM) and ScInfoVAE(SP) denote clustering of latent embedding features using the K-means algorithm and Spectral clustering algorithm respectively, while ScInfoVAE(RSC) denotes clustering using the RSC spectral clustering method. The results depicted in Fig. 2 show that the method proposed in this paper performs no worse than other clustering techniques on realistic datasets.

**Fig. 3:**
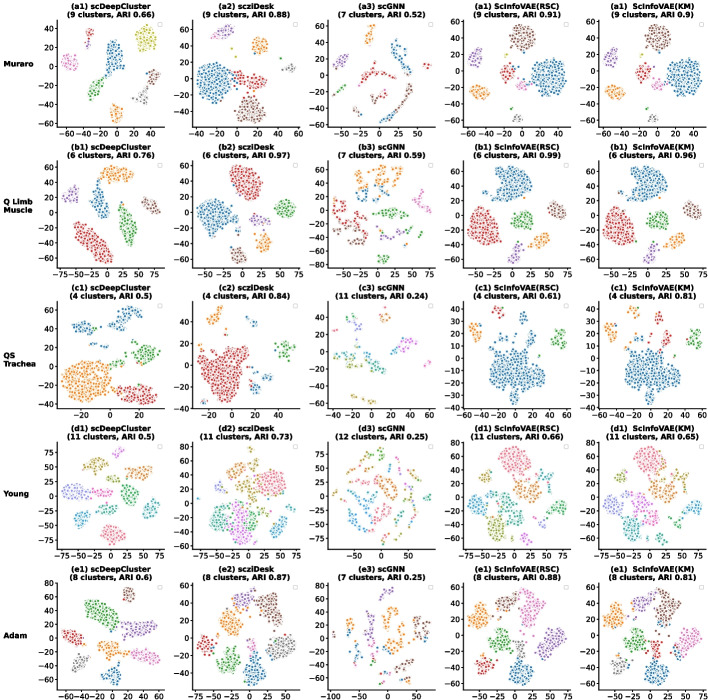
Visualization of identified clusters. The partitions identified with scDeepCluster, scziDesk, scGNN, ScInfoVAE(RSC) and ScInfoVAE(KM)) on five datasets (Muraro, Quake Limb Muscle, Quake Smart seq2 Trachea ,Young, Adam). The plots illustrate the t-SNE 2D projections of the created embeddings. All selected methods start by producing an embedding for the cells, which is clustered in a second phase. The quality of the method depends on both the created embedding and the clustering algorithm. Both our methods clustered the same embedding, produced by ScInfoVAE

Figure 3 visualized the results on five real data sets. First, the embedding data obtained from the training of multiple models were t-SNE 2D projected and the clusters predicted by ScInfoVAE were compared with other competitive methods scziDesk, scDeepCluster and scGNN. Figure 3 shows that the clustering results calculated using ScInfoVAE are aligned with the given class labels, as well as forming well-defined cluster divisions.

All our experiments present the results of three consecutive runs of each method on each dataset. Our methods are usually highlighted in bold. This experimental setting is used to benchmark the presented methods on a collection of 15 real scRNA-seq datasets, as detailed in Supplementary Fig. 3-5. From the bar chart, it can be seen that many indicators have achieved the top 3 results.

#### Method stability analysis

**Fig. 4:**
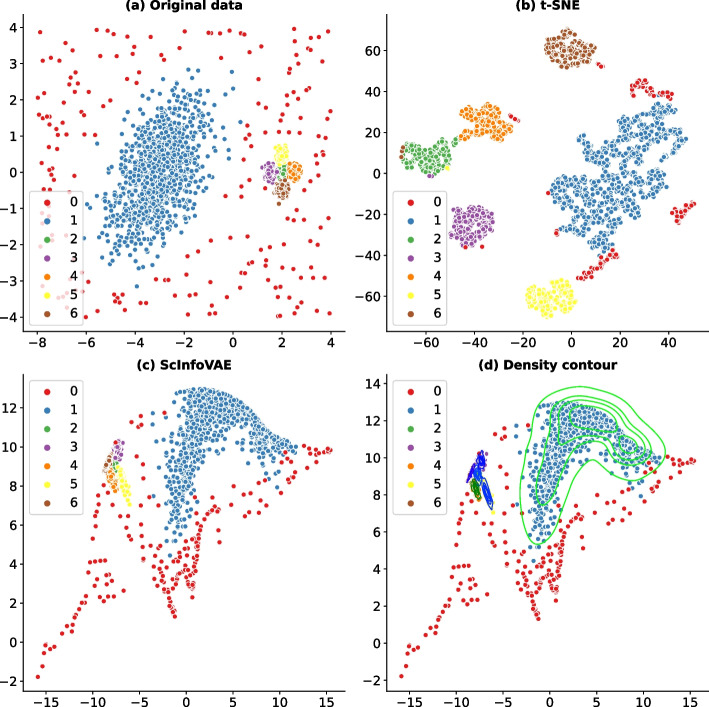
Benchmarking ScInfoVAE and t-SNE on synthetic data

The coefficient of variation (CV) is a relative measure of variability that indicates the size of a standard deviation in relation to its mean, calculated as standard deviation divided by mean score, it allows to compare the stability of results across values having potentially different ranges for each dataset (Fig. 4a-d). All analyzed methods have been executed 3 times on each dataset, starting from different initialization seeds.The results suggest that ScInfoVAE(KM) has an average stability compared to the other competing methods, while the most similar techniques, scziDesk, scDeepCluster and scGNN are generally more unstable. Clustering the learned embedding with K-means or with RSC does not change the stability of the predicted partition.

Two known shortcomings of VAEs are that (i) The variational bound (ELBO) can lead to poor approximation of the true likelihood and inaccurate models and (ii) the model can ignore the learned latent representation when the decoder is too powerful. InfoVAE is proposed to tackle these problems by adding an explicit mutual information term to the standard VAE objective. InfoVAE with three different divergences: Jensen, Stein Variational Gradient and Maximum-Mean Discrepancy. Results seem to indicate that InfoVAE leads to more principled latent representations, and better balance between reconstruction and latent space usage. As such, reconstructions might not look as crisp than ones from VAE but unsupervised task that make use of the representation are of better clustering. As shown in Supplementary Fig. 9, for the 5 real dataset experimentation, it show that InfoVAE extracts better latent representation for clustering than VAE, ARI clustering results are somewhat enhanced.

#### Computational complexity analysis

Available data in machine learning applications is becoming increasingly complex, due to higher dimensionality and difficult classes. There exists a wide variety of approaches to measuring complexity of labeled data, according to class overlap, separability or boundary shapes, as well as group morphology. For the original data before dimensionality reduction, many traditional clustering methods run and consume large amounts of memory and fail. For example, the spectral-based clustering methods (RSC) were imposed with quadratic space complexity, and we failed to run them with even large memory (for example, 256G). Many autoencoder based clustering techniques can transform the original input data and batch input in order to specifically reduce memory overhead.

After having analyzed the clustering performance of ScInfoVAE , the average execution time across all real-world scRNA-seq datasets is presented in Supplementary Fig. 8. For each real dataset, each method has been executed 3 times. The scores reported by our methods are average execution times, being placed at the median of all analyzed methods. All methods have been executed on GPU.The computational efficiency is explained by the short convergence times (10 epochs for graph-sc) while for instance, scziDesk and scDeepCluster perform a pretraining of 1000 and respectively 600 epochs, In ScInfoVAE, the epoch is not adjusted according to the data set size , it is all perform a pretraining of 1000 and a funetraining of 1000, it won’t stop finetraining early as scziDesk when gradient don’t descent, time overhead is greater than other methods based on neural networks. Therefore, adjusting the epoch parameter can reduce the time overhead of ScInfoVAE. Using only the most variable genes as input cell representations, instead of the usual high dimensional space brings a computational gain, reduces computing time. ScDeepCluster using original data as input , run time is much longer than other methods.

#### Experimental results

As in reference [28], to demonstrate that ScInfoVAE is able to robustly learn low-dimensional representations of the input data, we first simulated the data in two dimensions (for visualization purposes), as shown in Fig. 4a. The large cluster on the left consists of 1000 points and the five smaller clusters on the right each have 200 points. These five small clusters are very close to each other and can be roughly considered as one large cluster. Around these six clusters are 200 uniformly distributed outlier points. Each two-dimensional data point with coordinates (*x*, *y*), for the reason that nine-dimensional space occurs frequently in mathematics, and is a perfectly legitimate construct. Then for each two-dimensional data point with coordinates (x, y), we map it to a nine-dimensional space by the transformation $(x + y, x - y, xy, x^2, y^2, x^2 y, xy^2, x^3, y^3)$. Each of the nine features is then divided by its corresponding maximum absolute value.

Figure 4a shows the original 2200 two-dimensional synthetic data points (they are labeled by the clustering labels). Randomly distributed outliers are also indicated by different colors. In general, the two-dimensional representation of ten runs (Supplementary Fig. 6 ScInfoVAE 1-6) shows a similar pattern to that in Fig. 4c. As a comparison, we also ran t-SNE six times and the results (Supplementary Fig. 6 t-SNE 1-6) show that the layout of the clusters is not preserved as in Fig. 4b, e.g. the relative positions of the clusters change from run to run. Thus, by obtaining low-dimensional density information (Fig. 4d), we can better explain the structure in the original data and account for the uncertainty in the predictions.

**Fig. 5:**
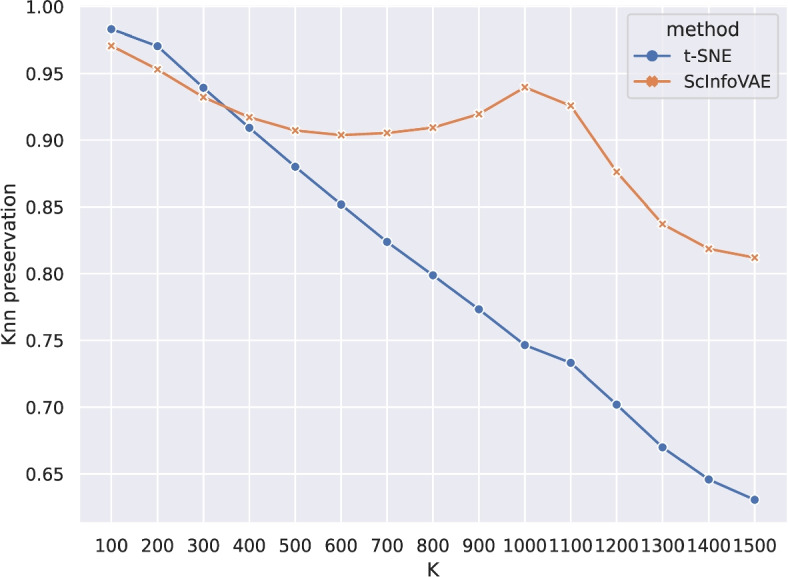
Average K-nearest neighbor preservations across ten runs for different Ks

High-dimensional data is an integral part of modern systems biology. Computational methods for dimensional reduction are rapidly evolving for application to single-cell and multimodal techniques. To understand the impact of these non-linear transformations on the underlying biological patterns in our data, we tested K-nearest neighbor preservation before and after the transformations using the DR algorithm [29]. We then calculated the K-nearest neighbor preservation values ($K = 100$ to 1500) and found that the ScInfoVAE results had significantly higher Knn preservation values than the t-SNE results, as shown in Fig. 5.

**Fig. 6:**
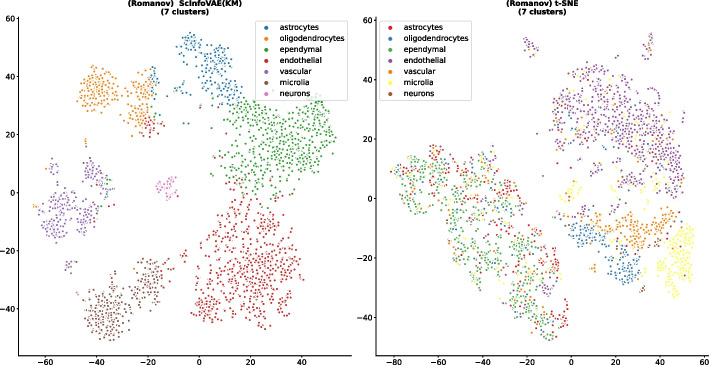
Average K-nearest neighbor preservations across ten runs for different Ks

To demonstrate that ScInfoVAE leads to the interpretability of the model, A scatter plot is presented in the Fig. 6, illustrating the cell distribution obtained by training the ScInfoVAE model on the Romanov dataset [30] on the left, and by training t-SNE on the right. The cells in the plots are categorized into seven main types: oligodendrocytes, neurons, astrocytes, ependymal cells, endothelial cells, vascular lineage, and microglia. we observed a close proximity between ependymal and endothelial cells, while ependymal cells were farther from oligodendrocytes and neurons. This observation can be explained by the distinct gene expression patterns and biological functions of these cell types, which are captured by our method. Ependymal and endothelial cells both play important roles in maintaining the brain’s microenvironment. Ependymal cells are involved in the production and circulation of cerebrospinal fluid (CSF), whereas endothelial cells contribute to the formation of the blood-brain barrier (BBB). The shared functions of these cell types in regulating the brain’s extracellular milieu likely lead to similarities in their gene expression patterns, which our method detects and represents through their proximity in the graph. Conversely, oligodendrocytes and neurons have different functions and gene expression patterns compared to ependymal cells. Oligodendrocytes are responsible for producing myelin, which insulates axons, while neurons serve as the primary information-processing cells in the brain. These differences in function and gene expression result in their separation from ependymal cells in the graph, highlighting the ability of our algorithm to identify and distinguish between diverse cell types based on their gene expression profiles. In contrast, t-SNE can also differentiate between different cell types, but the scatter plot suggests that a significant number of cells are intertwined in the figure, including ependymal and endothelial cells, as well as neurons and the other six types of cells.

## Conclusion

In this paper, we propose a new method, ScInfoVAE, which combines the InfoVAE depth model with a zero-inflated negative binomial distributed model for clustering scRNA-seq data. The proposed method produces competitive results on real data sets, and the low-dimensional representation learned by ScInfoVAE preserves local and global neighbourhood structure data well. We believe that this advantage of the framework can open up new research directions and improve the clustering results of existing models. However, due to the diversity of single-cell data distributions, improvements to model performance may not always be readily achievable in this case. We hope that this work will inspire future researchers considering mutual information models for the analysis of scRNA-seq.

## Supplementary Information

**Additional file 1.**

## Data Availability

The datasets analysed during the current study are available in the Github repository, “https://github.com/ttgump/scDeepCluster/tree/master/scRNA-seq%20data”, and Drive Google, “https://drive.google.com/drive/folders/1BIZxZNbouPtGf_cyu7vM44G5EcbxECeu”.
