# Supplementary material for: ScInfoVAE: interpretable dimensional reduction of single cell transcription data with variational autoencoders and extended mutual information regularization
Source: BioData Min. 2023 Jun 10;16:17. doi: 10.1186/s13040-023-00333-1 (PMC10257850; doi:10.1186/s13040-023-00333-1)
Supplement: Supplementary file 1 — Additional file 1. [file 13040_2023_333_MOESM1_ESM.docx]

ScInfoVAE:Interpretable dimensional reduction of single cell transcription data with variational autoencoders and extended mutual information regularization

Weiquan Pan , Faning Long , Jian Pan

**Supplementary Materials**

Table 1 The list of scRNA-seq datasets used in this study

| **Dataset name** | **platform** | **Classes** | **cells** | **genes** | **Sparsity** | **Ref.** |
| --- | --- | --- | --- | --- | --- | --- |
| Adam | Drop-seq | 8 | 3660 | 23797 | 92.33% | [1] |
| Muraro | CEL-seq2 | 9 | 2122 | 19046 | 73.02% | [2] |
| Quake Smart seq2 Diaphragm | Smart-seq2 | 5 | 870 | 23341 | 91.35% | [3] |
| Quake Smart seq2 Limb Muscle | Smart-seq2 | 6 | 1090 | 23341 | 89.47% | [3] |
| Quake Smart seq2 Lung | Smart-seq2 | 11 | 1676 | 23341 | 89.08% | [3] |
| Quake Smart seq2 Trachea | Smart-seq2 | 4 | 1350 | 23341 | 85.48% | [3] |
| Quake10x Bladder | 10x | 4 | 2500 | 23341 | 86.94% | [3] |
| Quake10x Limb Muscle | 10x | 6 | 3909 | 23341 | 93.57% | [3] |
| Quake10x Spleen | 10x | 5 | 9552 | 23341 | 94.34% | [3] |
| Romanov | unknown | 7 | 2881 | 21143 | 85.92% | [4] |
| Worm neuron cell | scRNA-seq | 10 | 4186 | 13488 | 98,61% | [5] |
| Mouse ES cells | Droplet barcoding | 4 | 2717 | 20670 | 65,76% | [6] |
| Mouse bladder cell | Microwell-seq | 16 | 2746 | 20670 | 94,86 % | [7] |
| 10x PBMC | 10X | 8 | 4271 | 16653 | 92,23% | [8] |
| Young | W | 11 | 5685 | 33658 | 94.70% | [9] |

Table 2. Accessibility of benchmaked methods and the programming language of the implementation

|  | **Method** | **Requires nb clusters** | **Programming language** | **Availability** |
| --- | --- | --- | --- | --- |
| 1 | PCA + KMeans | yes | python | https://scikilearn.org/stable/modules/generated/sklearn.decomposition.PCA.html  https://scikitlearn.org/stable/modules/generated/sklearn.cluster.KMeans.html |
| 2 | scDeepClustering | yes | python | https://github.com/ttgump/scDeepCluster |
| 3 | scziDesk | yes | python | https://github.com/xuebaliang/scziDesk |
| 4 | scanpy/Seurat | no | Python | https://scanpy.readthedocs.io/en/stable/tutorials.html#clustering |
| 5 | desc | no | Python | https://github.com/eleozzr/desc |
| 6 | scRNA | yes | Python | https://github.com/nicococo/scRNA |
| 7 | scedar | no | Python | https://scedar.readthedocs.io/en/latest/index.html |
| 8 | cidr | yes | R | https://github.com/VCCRI/CIDR |
| 9 | soup | yes | R | https://rdrr.io/github/lingxuez/SOUP/ |
| 10 | scvi | no | R | https://github.com/YosefLab/scvi-tools |
| 11 | raceid | no | R | https://cran.rproject.org/web/packages/RaceID/vignettes/RaceID.html |
| 12 | scGNN | no | Python | https://github.com/juexinwang/scGNN |


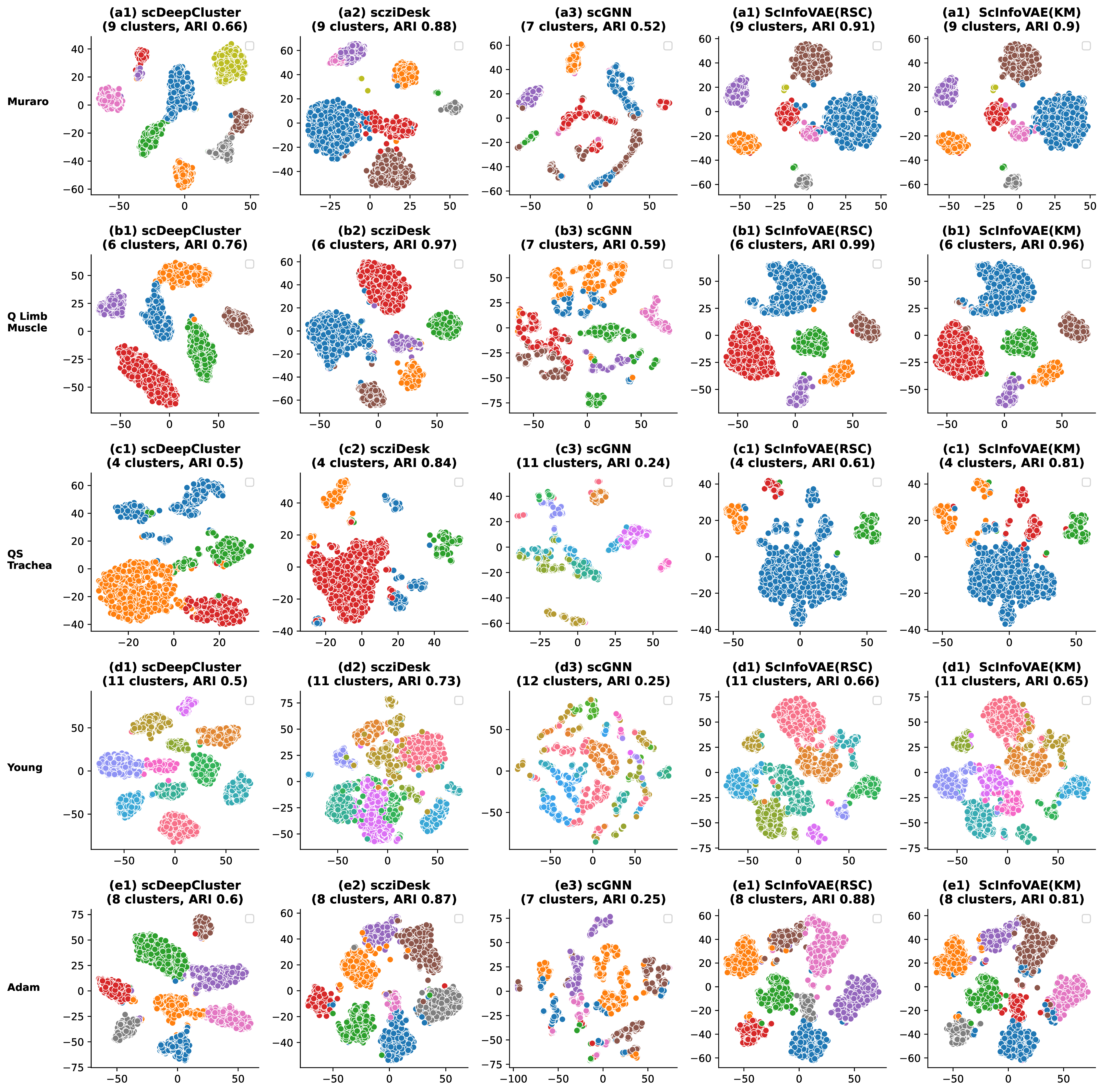


Supplementary Fig.1. Visualization of identified clusters. The partitions identified with scDeepCluster, scziDesk, scGNN, ScInfoVAE(RSC) and ScInfoVAE(KM). The plots illustrate the t-SNE 2D projections of the created embeddings. All selected methods start by producing an embedding for the cells, which is clustered in a second phase. The quality of the method depends on both the created embedding and the clustering algorithm. Both our methods clustered the same embedding, produced by ScInfoVAE.


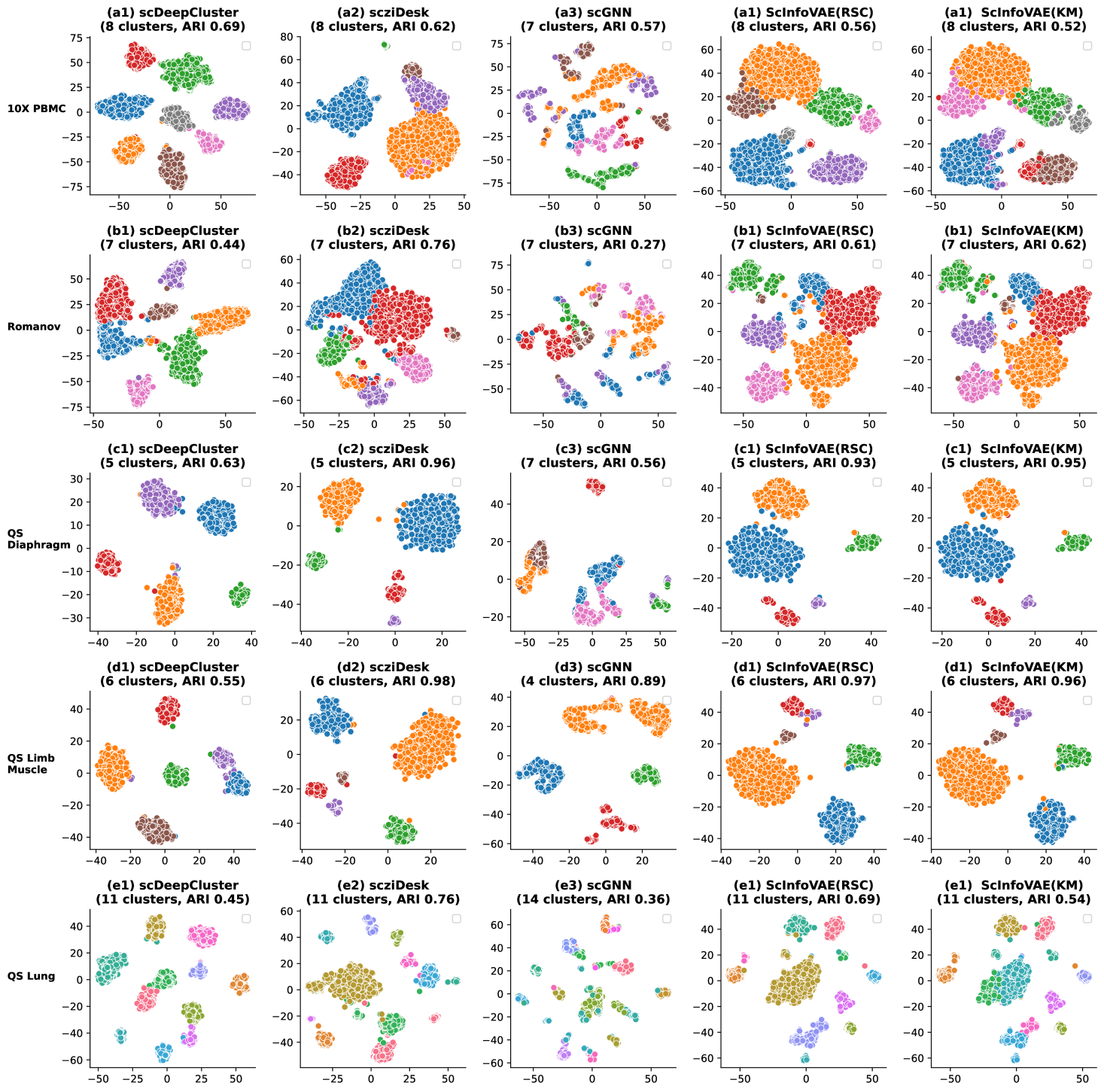


Supplementary Fig. 2. Visualization of identified clusters. The partitions identified with scDeepCluster, scziDesk, scGNN, ScInfoVAE(RSC) and ScInfoVAE(KM). The plots illustrate the t-SNE 2D projections of the created embeddings. All selected methods start by producing an embedding for the cells, which is clustered in a second phase. The quality of the method depends on both the created embedding and the clustering algorithm. Both our methods clustered the same embedding, produced by ScInfoVAE.


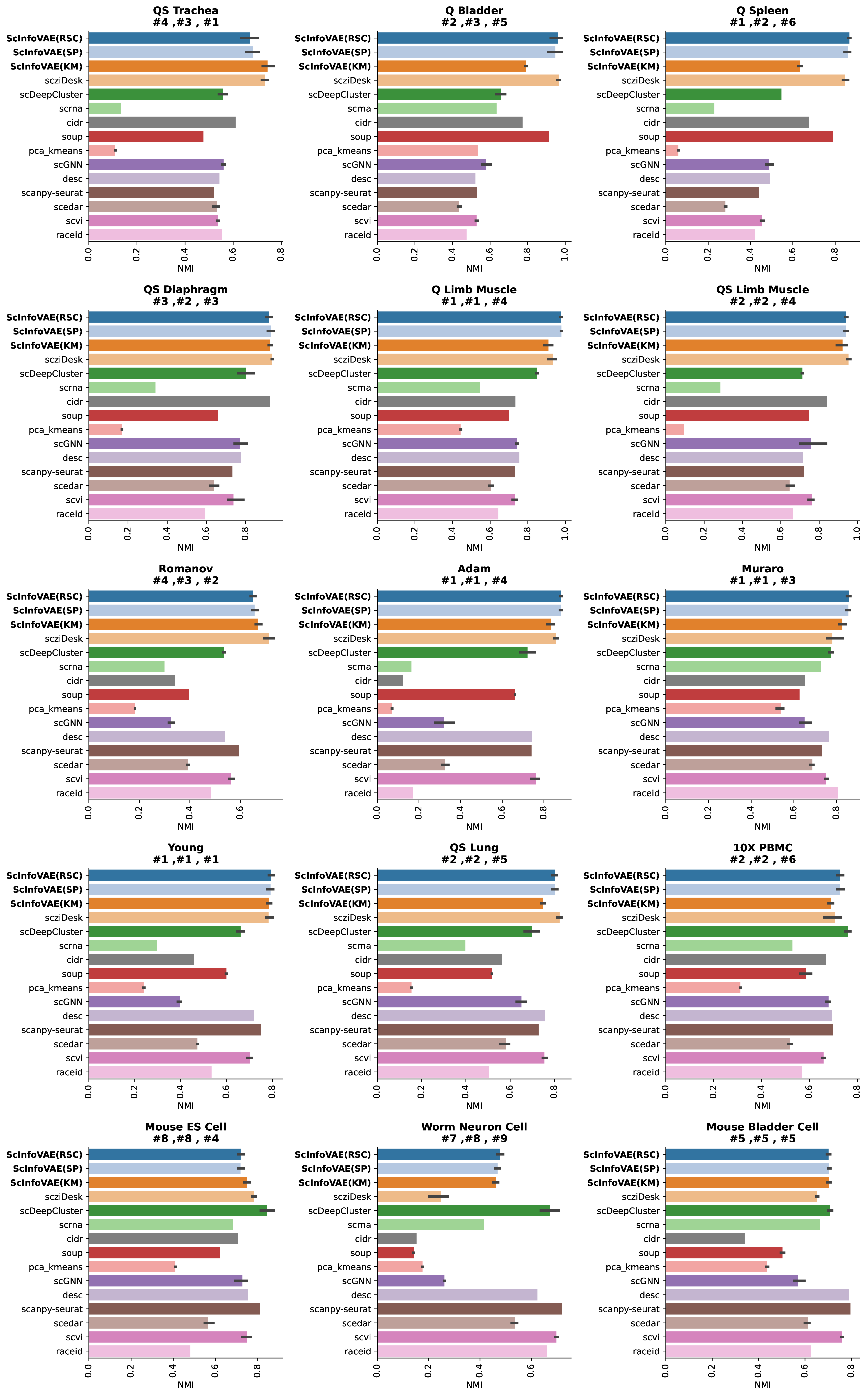


Supplementary Fig. 3. Dataset-level analysis of real scRNA-seq data on NMI scores. The dataset annotations (e.g. #1) indicate the ranking of graph-sc, respectively, with K-means and RSC clustering on each analyzed dataset


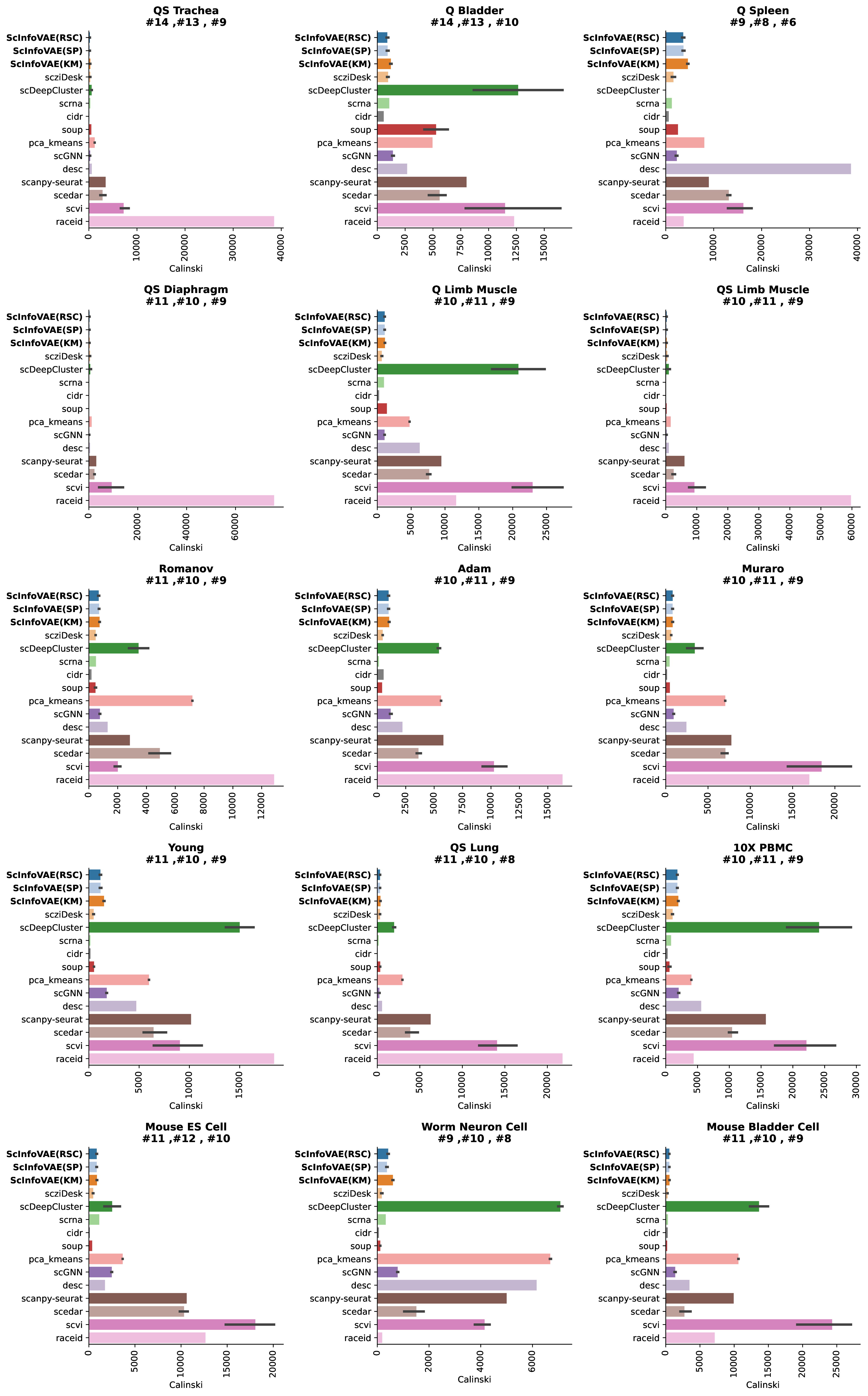


Supplementary Fig. 4. Dataset-level analysis of real scRNA-seq data on Calinski scores. The dataset annotations (e.g. #1) indicate the ranking of graph-sc, respectively, with K-means and RSC clustering on each analyzed dataset


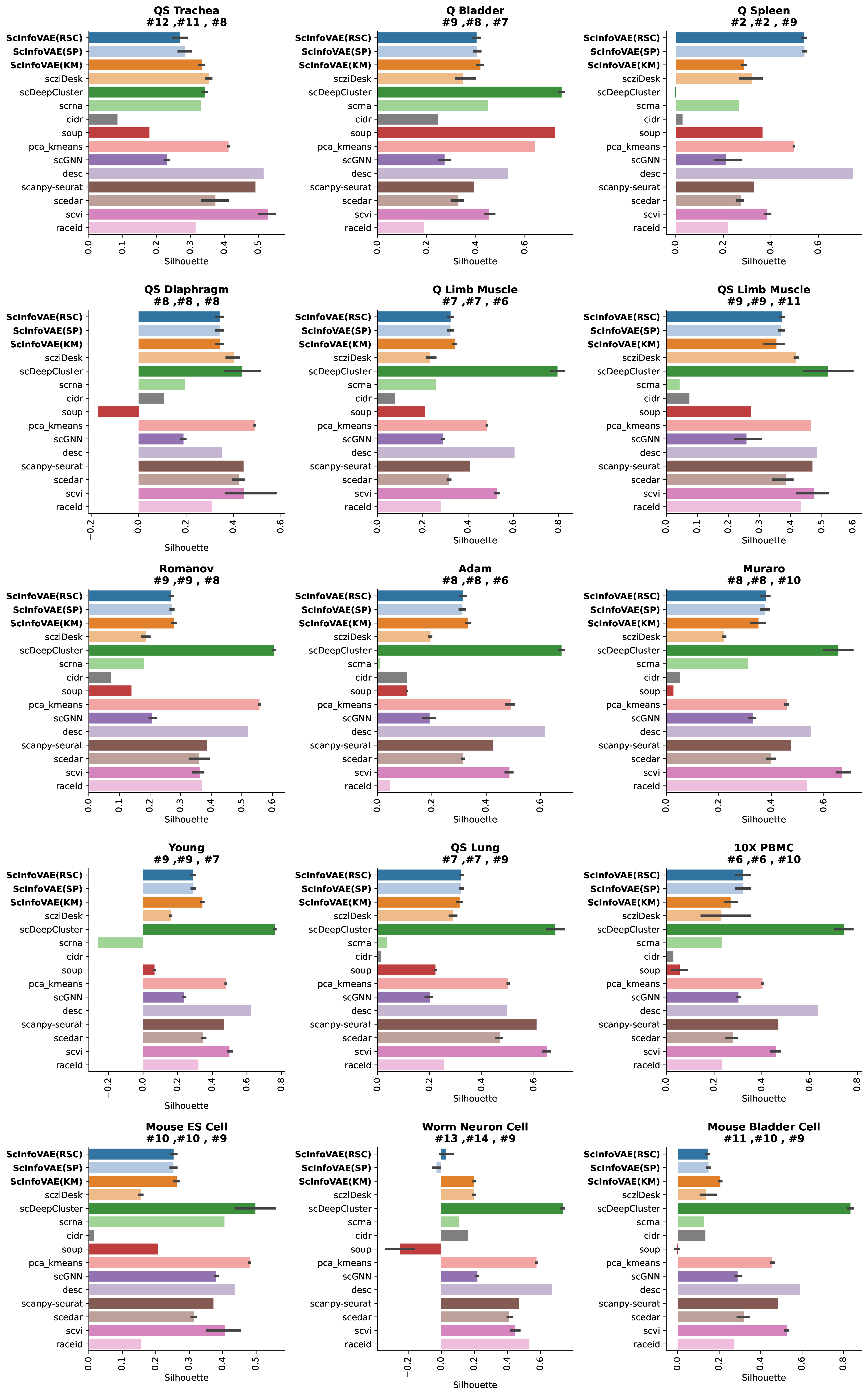


Supplementary Fig. 5. Dataset-level analysis of real scRNA-seq data on Silhouette scores. The dataset annotations (e.g. #1) indicate the ranking of graph-sc, respectively, with K-means and RSC clustering on each analyzed dataset


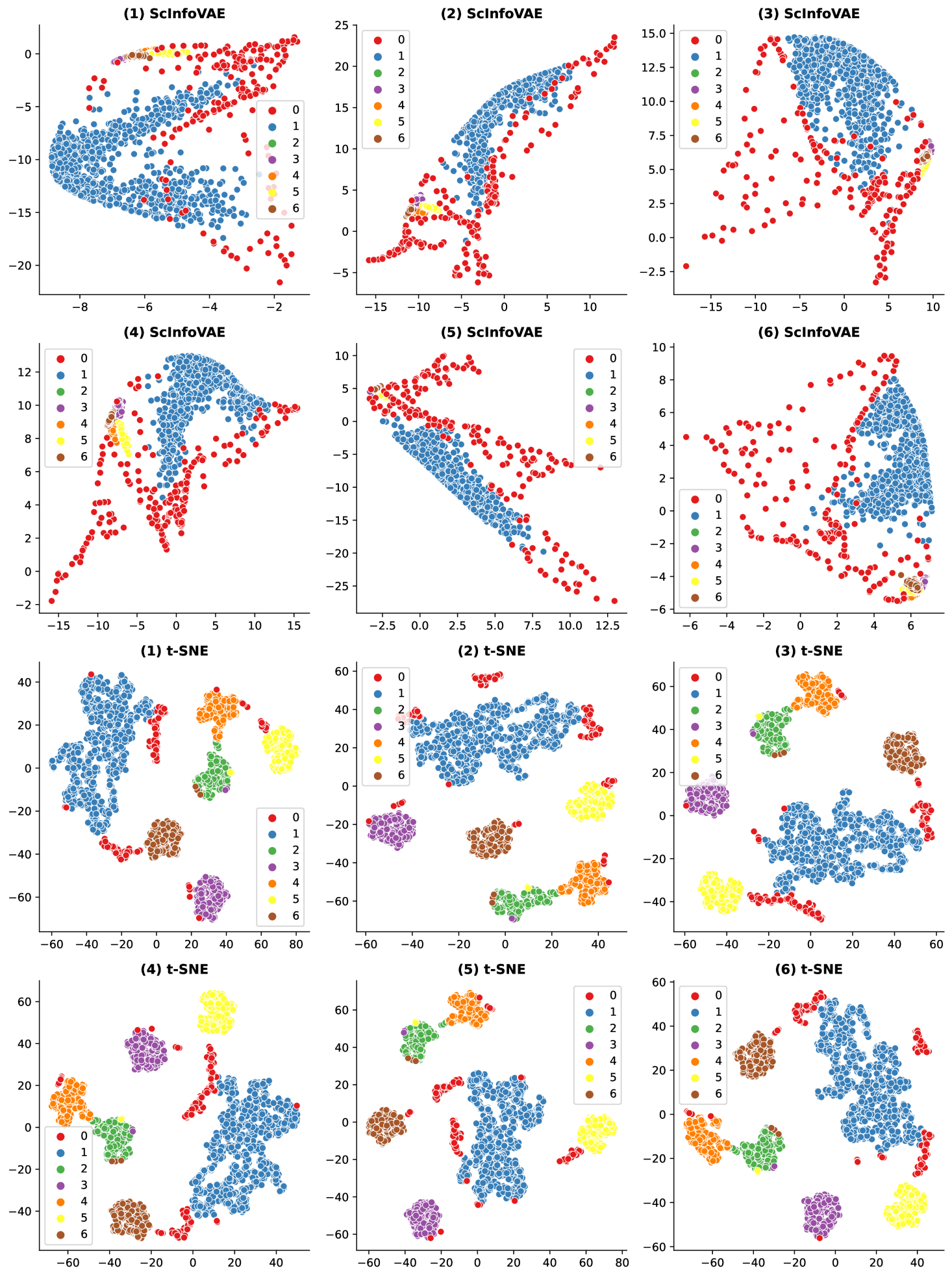


Supplementary Fig. 6: Repeated six runs on the simulated nine-dimensional synthetic dataset (2,200 data points). SCInfoVAE results, and t-SNE results. Here the color and symbol combinations encode clusters.


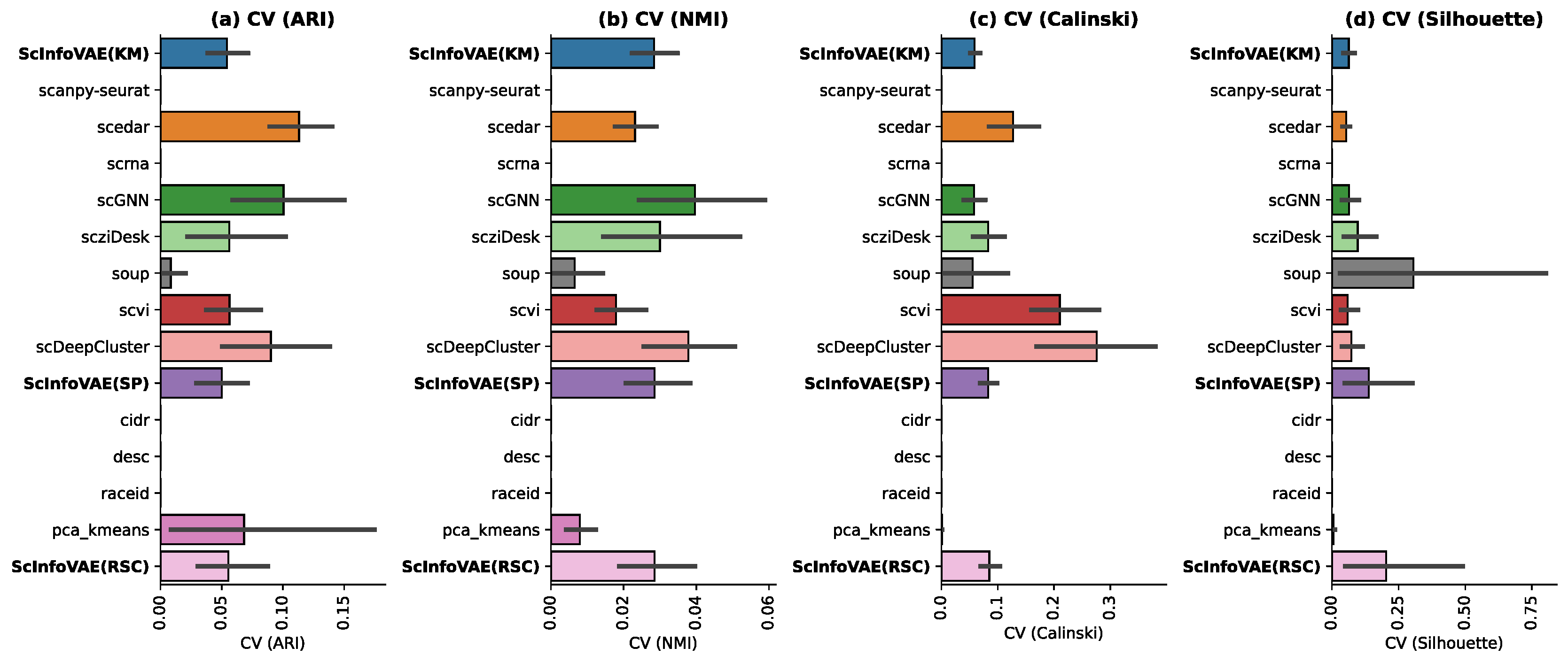


Supplementary Fig. 7 The model stability (a-d) across the three runs on the real-world datasets has been depicted as the coefficient of variation (for each dataset, the standard deviation of the runs is divided by the average result). Here, the lower the score, the more stable the model.


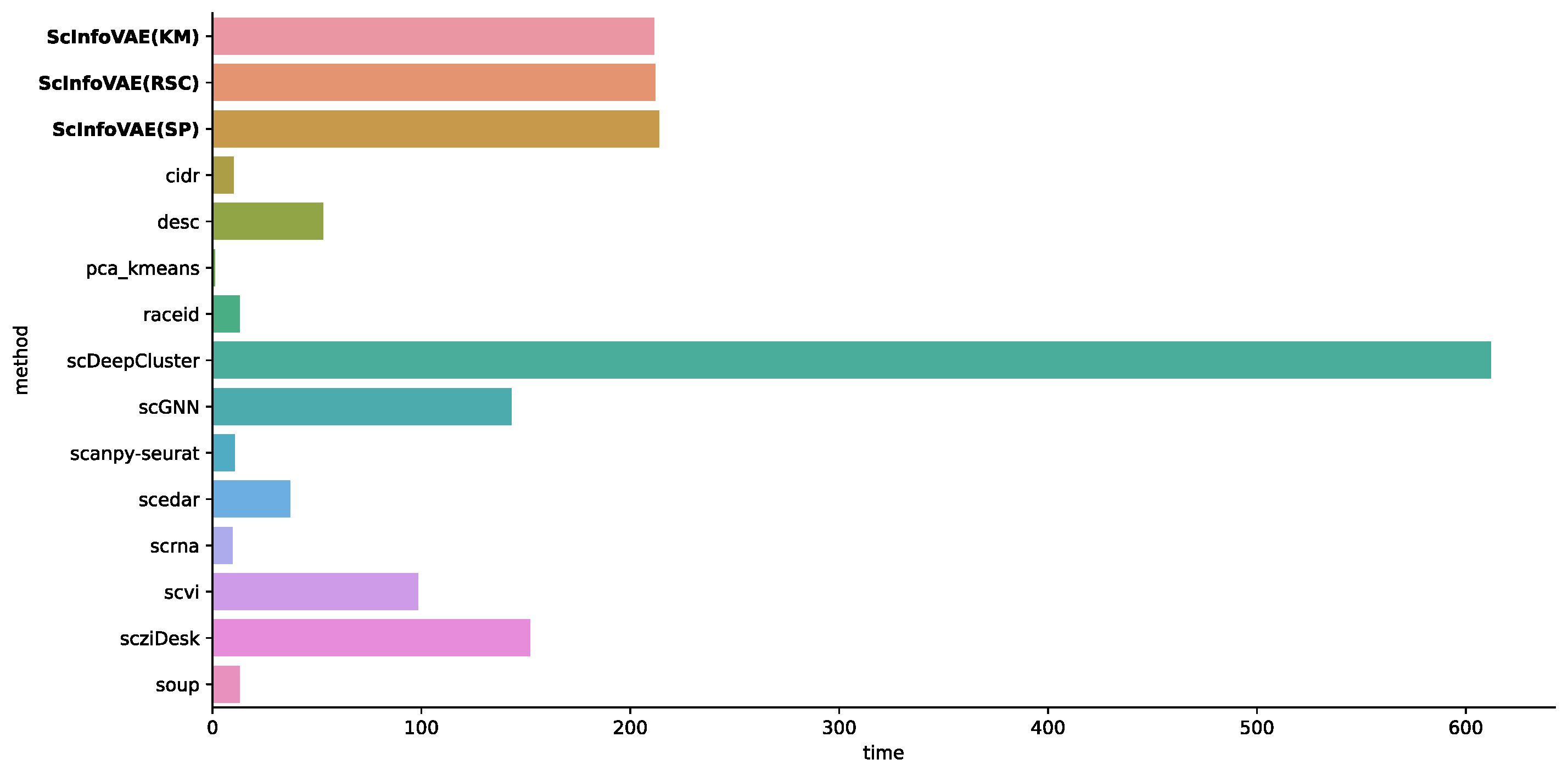


Supplementary Fig. 8 Execution time and scalability analysis. Average execution time for all benchmarked methods


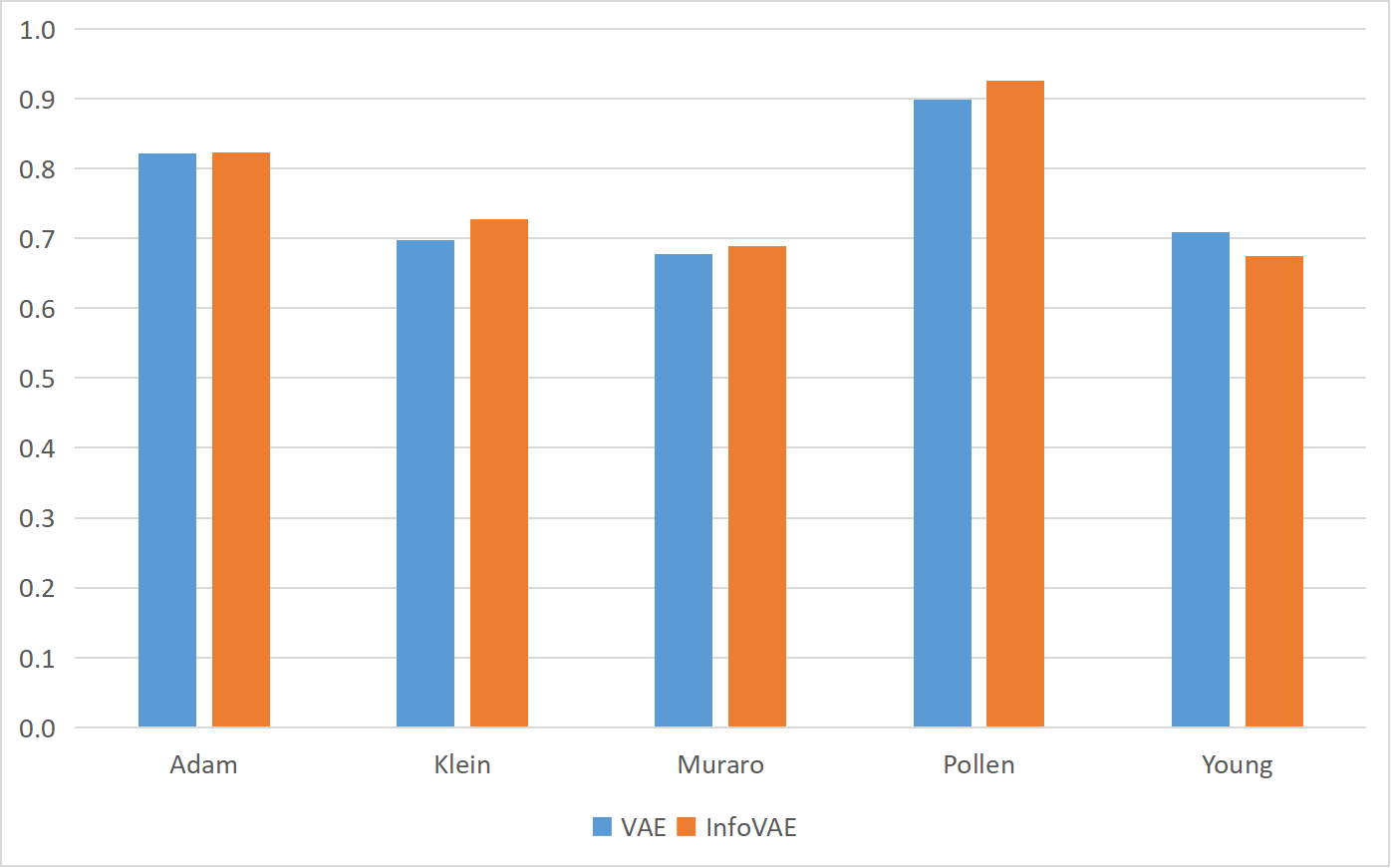


Supplementary Fig. 9 VAE and InfoVAE extracts latent representation, compare Kmeans clustering ARI result In 5 real dataset

**References**

1. Adam M, Potter AS, Potter SS. Psychrophilic proteases dramatically reduce single cell RNA-seq artifacts: a molecular atlas of kidney development. Development 2017;144(19): 3625-3632.
2. Muraro MJ, Dharmadhikari G, Gr¨un D, Groen N, Dielen T, Jansen E, et al. A single-cell transcriptome atlas of the human pancreas. Cell Syst 2016; 3(4): 385-394
3. Schaum N, Karkanias J, Neff NF, May AP, Quake SR, Wyss-Coray T, et al. Singlecell transcriptomics of 20 mouse organs creates a Tabula Muris: The Tabula Muris Consortium. Nature 2018; 562(7727): 367.
4. Romanov RA, Zeisel A, Bakker J, Girach F, Hellysaz A, Tomer R, et al. Molecular interrogation of hypothalamic organization reveals distinct dopamine neuronal subtypes. Nat Neurosci 2017; 20(2): 176.
5. . Cao, J. et al. Comprehensive single-cell transcriptional profling of a multicellular organism. Science 357, 661–667 (2017).
6. Klein, A. M. et al. Droplet barcoding for single-cell transcriptomics applied to embryonic stem cells. Cell 161, 1187–1201 (2015).
7. Han, X. et al. Mapping the mouse cell atlas by Microwell-seq. Cell 172, 1091–1107 (2018).
8. Zheng, G. X. et al. Massively parallel digital transcriptional profling of single cells. Nat. Commun. 8, 14049 (2017).
9. Young MD, Mitchell TJ, Braga FAV, Tran MG, Stewart BJ, Ferdinand JR, et al. Single-cell transcriptomes from human kidneys reveal the cellular identity of renaltumors. Science 2018; 361(6402): 594-599.
